# Vascular Involvement of Immunoglobulin G4-Related Disease: A Case Report

**DOI:** 10.7759/cureus.37893

**Published:** 2023-04-20

**Authors:** Maan Jamjoom, Bsaim A Altirkistani, Yasir A Alghamdi, Ammar Y Alansari

**Affiliations:** 1 Emergency Medicine, King Abdullah International Medical Research Center, Jeddah, SAU; 2 College of Medicine, King Saud Bin Abdulaziz University for Health Sciences, Jeddah, SAU; 3 Emergency Medicine, Ministry of National Guard-Health Affairs, Jeddah, SAU

**Keywords:** steroids, heterogonous, vascular system, aortitis, igg4-related disease

## Abstract

Immunoglobulin-G4-related disease (IgG4-RD) is a fibro-inflammatory condition that can impact any organs/tissues, including the vascular systems, resulting in aortitis/periaortitis/periarteritis (PAO/PA). The complex nature of this disease and limited understanding have led to potential delays in identifying and managing irreversible organ damage. Herein, we report a 17-year-old female with hyper IgG4 disease, sclerosing mesenteritis, short stature, and insulin resistance who presented with symptoms of fever, epigastric pain, left flank pain, vomiting, dizziness, decreased urine output, and diarrhea. Imaging studies revealed an arterial wall thickening of the ascending aorta and aortic arch, splenic abscesses, and enlarged lymph nodes, consistent with IgG4-related aortitis. Treatment with steroids and antifungal agents was initiated. However, the patient developed septic shock and multi-organ failure requiring inotropes and mechanical ventilation. Ascending aortic aneurysm rupture, in this case, probably led to the patient's demise, but unfortunately, no autopsy was done to confirm it. This case highlights the importance of identifying and addressing vascular involvement in IgG4-RD to prevent irreversible organ damage and mortality.

## Introduction

Immunoglobulin G4-related disease (IgG4-RD) is a heterogeneous fibro-inflammatory condition in which it is characterized by hyper IgG4 serum levels and lymphoplasmacytic infiltrations of the involved organs, which can affect any organ or tissue, including the vascular system. For example, the aorta, iliac artery, and carotid artery are the commonly involved blood vessels in IgG4-related diseases resulting in conditions like aortitis/periaortitis/periarteritis (PAO/PA) [1]. Although the prevalence of vascular involvement in IgG4-related diseases ranges from 10-30%, the complex nature of this disease and limited understanding have led to potential delays in identifying and managing irreversible organ damage [1-2]. However, the lack of larger studies in such heterogenous conditions might contribute to potential delays in recognizing, managing, and even minimizing its irreversible organ damage [1].

## Case presentation

A 17-year-old female known case of hyper IgG4 disease, sclerosing mesenteritis, short stature, and insulin resistance since childhood presented to the emergency department complaining of fever, epigastric pain, left flank pain, vomiting, dizziness, decreased urine output, and diarrhea for five days. She has a negative history of sore throat, cough, runny nose, or chest pain. On bedside clinical assessment, she was conscious with mild respiratory distress and exhibited signs of septic shock; her initial vitals were blood pressure of 97/50, heart rate of 138, temperature of 38.8° C, respiratory rate of 28, and oxygen saturation of 100% on room air.

**Figure 1:**
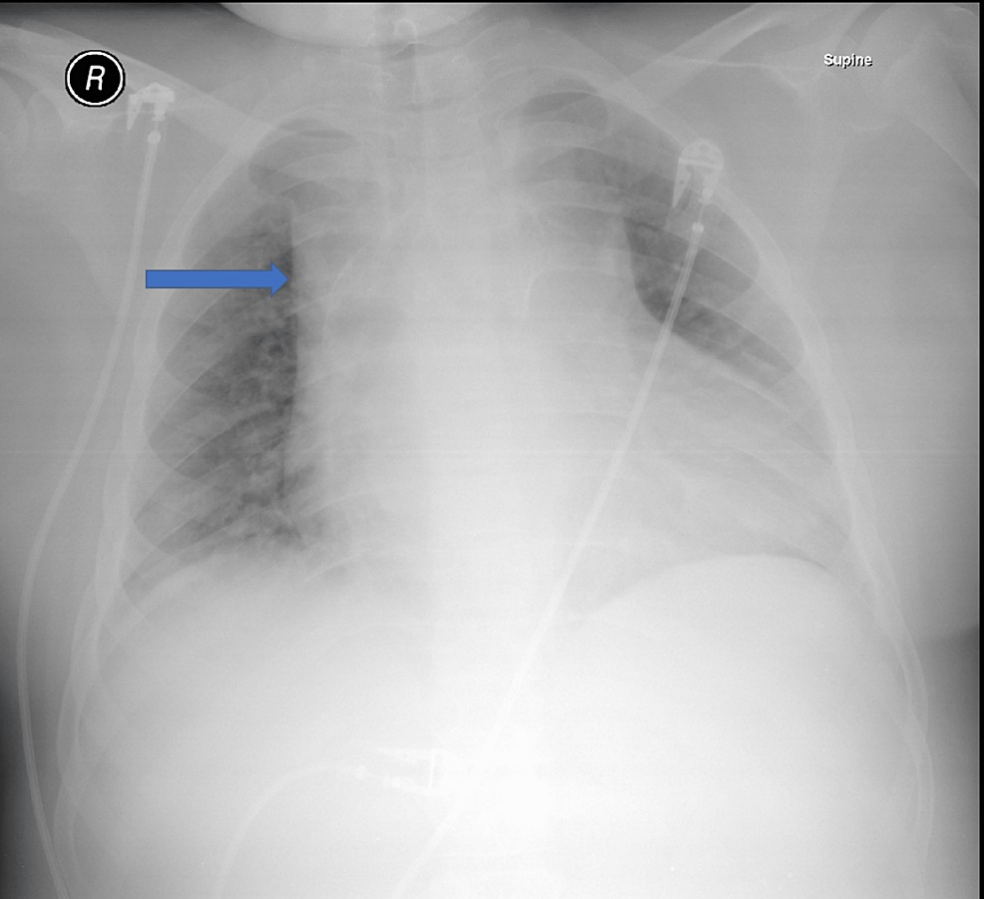
Portable X-ray of the chest

**Figure 2:**
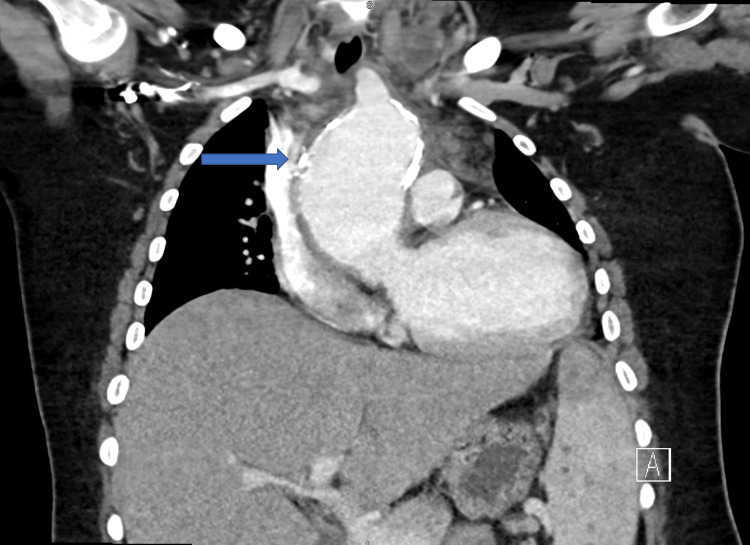
Coronal CT of the chest

**Figure 3:**
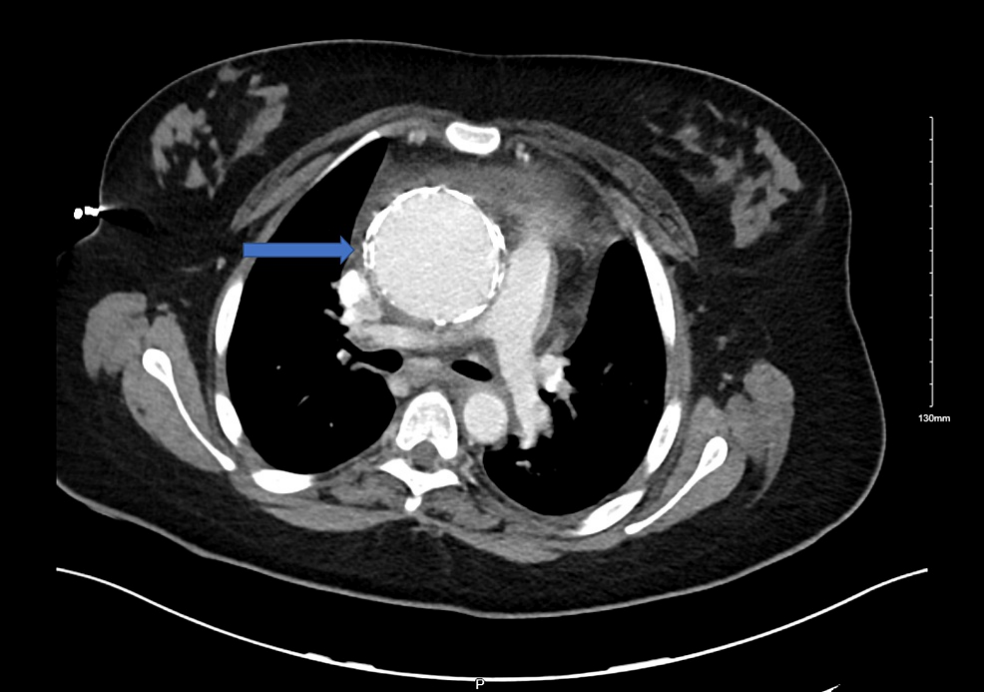
Axial CT of the chest

C-reactive protein and sedimentation rate were elevated. Chest X-ray was congested with wide mediastinum and left pleural effusion, as shown in Figure 1. The chest computed tomography (CT) showed arterial wall thickening of the ascending aorta and aortic arch not involving the aortic root with a diameter of 6.1 cm and extending to the level of the origin of the brachiocephalic artery associated with the occluded left circumflex artery, as shown in Figure 2. It is documented that the risk of rupture was 15-20%. Rim of pericardial effusion and left pleural effusion were noted. Surrounding fat stranding and lymphadenopathy were also reported. Enlarged lymph nodes and lung nodules were likely to underlie IgG4 disease. Moreover, CT pulmonary angiogram revealed filling defects in the left posterior and lateral basal segmental pulmonary artery branches suggestive of acute pulmonary embolism (PE), as shown in Figure 3. The CT of the abdomen showed splenomegaly with suggestive abscesses. Echo revealed aortic valve sclerosis and moderate aortic regurgitation with a left ventricular ejection fraction of 42%. Transthoracic echo (TTE) and transesophageal echo (TEE) didn't reveal any vegetation. Furthermore, antinuclear antibodies, anti-double stranded DNA antibodies, antineutrophilic cytoplasmic antibodies, antiphospholipid antibodies, and rheumatoid factor antibodies were negative. Hepatitis, brucella, dengue, Epstein-Barr virus, varicella, cytomegalovirus, and syphilis serology were all negative. Possible diagnoses were mediastinitis, aortitis, or Takayasu's arteritis.

The patient was prescribed prednisolone 60 mg to control the inflammatory process and for the known response for IgG4 disease to steroids. She was advised to remain on this dose for three weeks and then gradually taper off. Anidulafungin was administered for a total of 10 days. For managing sepsis, meropenem and vancomycin were administered for a total of 14 days. Enoxaparin 80 mg bid was administered for PE. Surgical interventions for the aorta were not considered during the event due to the high risk of bleeding and rupture. Lastly, upon discharge, the patient was started on mycophenolate, vitamins C and D, alendronate, and oriented colchicine.

A few months later, the patient was admitted for two months with septic shock and multi-organ failure, coagulopathy, lactic acidosis, and significant hemoglobin drop. She was admitted to the intensive care unit with mechanical ventilation. Thereafter, she was extubated and shifted to bilevel positive airway pressure (BiPAP). This was followed by multiple attacks of septic shock and multi-organ failure, requiring inotropes and mechanical ventilation. TTE and TEE showed severely dilated left ventricle (LV) with an ejection fraction (EF) of 20% and global LV hypokinesia. Consequently, given her multi-organ failure and overall prognosis, she was designated as "not for resuscitation" care after a discussion with her family in the appropriate manner. The possibility of sudden death due to aortic rupture was highlighted in that delicate discussion. Nevertheless, the patient showed improvements in her renal functions and stabilization of her hemodynamics, leading to extubation to BiPAP. Unfortunately, the patient experienced sudden cardiac arrest in the form of pulseless electrical activity (PEA). The cause of death was likely due to the rupture of an ascending aortic aneurysm, as noted in the death report. No autopsy was done.

## Discussion

A decade ago, the first documented cases of vascular involvement in IgG4 disease emerged, with subsequent observations indicating a high prevalence of involvement in the abdominal and thoracic aorta, manifested as aortitis or periaortitis [3]. However, the term periarteritis was later emphasized to describe medium-large sized arteries affected by arterial wall thickening [4].

It has been shown that males were commonly found to have IgG4 aortitis compared to females [1]. The etiology of aortitis/periaortitis is not solely due to atherosclerosis but rather may involve autoimmune factors, lipoproteins, and certain haplotypes of human leukocyte antigens (HLA) [5-9]. Furthermore, inflammation markers were found to be elevated in IgG4 disease patients who had vascular involvement [1,10]. More so, half of the patients with parotitis or inflammatory abdominal aneurysms had positive antinuclear antibodies [11,12]. Obtaining histopathological results can be difficult in IgG4 disease; nevertheless, it involves other tissue or organs, which makes it possible for other biopsies from other sites [13,14].

Regarding treatments, vascular involvements in IgG4 disease were reported to be not an exception from the use of steroids [15]. However, a deterioration was observed after steroid use in some patients with involved vascular system defects. Therefore, lower doses and gradual tarping of steroids could be an option to consider alongside the use of immunosuppression, such as mycophenolate, azathioprine, or methotrexate [16].

## Conclusions

In conclusion, even though the clinical manifestations of vascular inflammations in IgG4 disease can be challenging and may result in delayed diagnosis, physicians should be vigilant and aware of the vascular involvements in such patients, as the commonly involved parts are the aorta followed by iliac and coronary arteries. Nevertheless, with new advanced techniques, it could contribute to rapid recognition and appropriate management of such cases.
